# Correction: A dual-clock-driven model of lymphatic muscle cell pacemaking to emulate knock-out of Ano1 or IP_3_R

**DOI:** 10.1085/jgp.20231335501222024c

**Published:** 2024-01-31

**Authors:** Edward J. Hancock, Scott D. Zawieja, Charlie Macaskill, Michael J. Davis, Christopher D. Bertram

Vol. 155, No. 12 | https://doi.org/10.1085/jgp.202313355 | October 18, 2023

The authors regret that, in the original article, parameters *K*_C_ and *q* were inadvertently omitted from Table 1. The revised table is presented here with the added rows. The error appears in print and in PDFs downloaded before January 22, 2024.

**Table 1.**
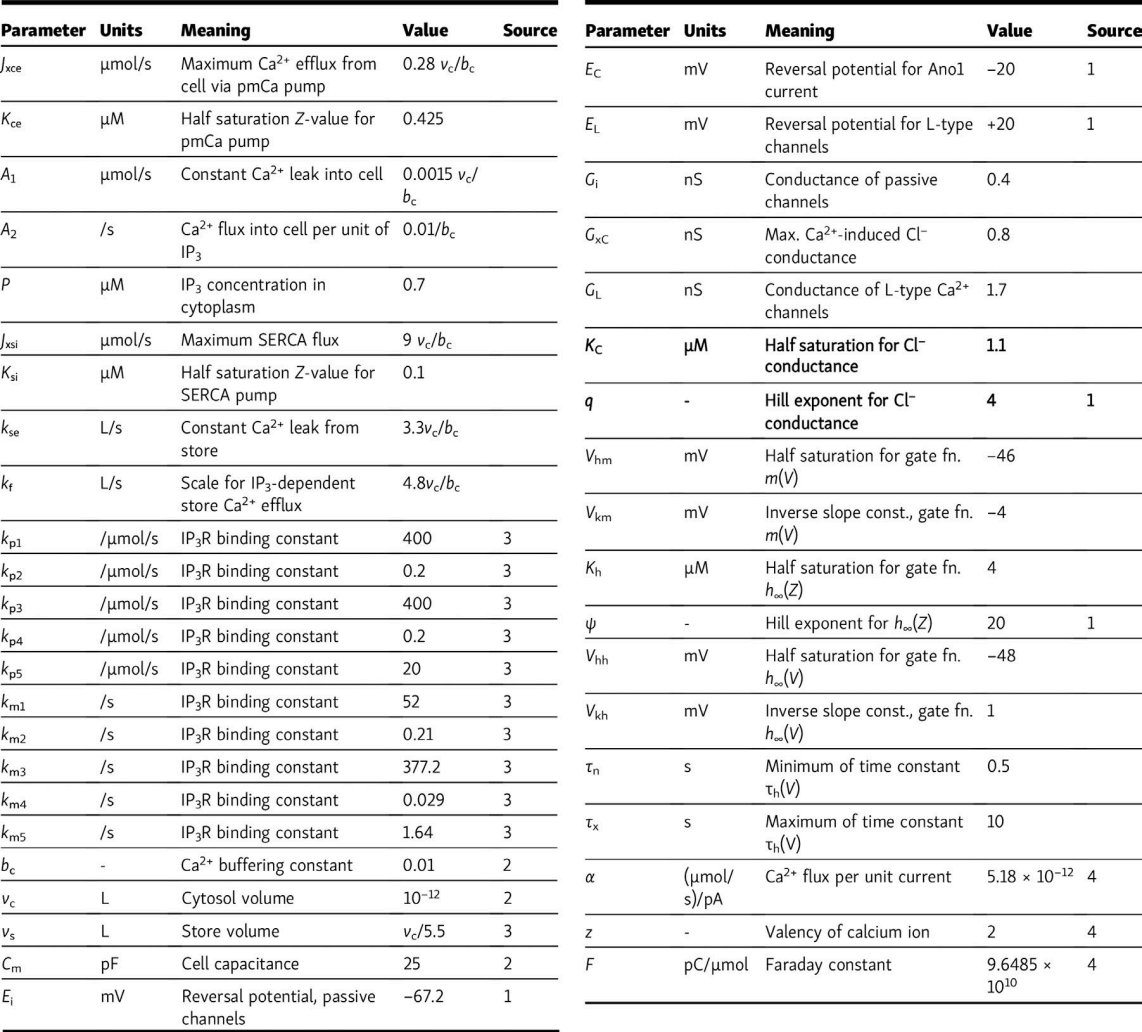
Values for constants in the model

Sources (otherwise, estimated): 1 is Imtiaz et al. (2007), although in many cases the values have been reinterpreted in terms of units, etc., 2 is Lees-Green et al. (2014), 3 is Keener and Sneyd (2009), and 4 is a universal physical constant. Estimated values were based on existing sources. L, litre.
